# Supplementary figures and images for: Integrated IBD Analysis, GWAS Analysis and Transcriptome Analysis to Identify the Candidate Genes for White Spot Disease in Maize
Source: Int J Mol Sci. 2023 Jun 11;24(12):10005. doi: 10.3390/ijms241210005 (PMC10298249; doi:10.3390/ijms241210005)

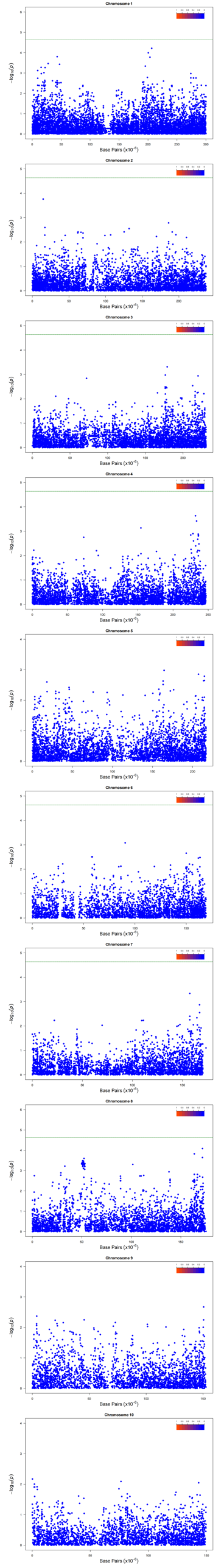

Supplement: Supplementary file 1 [file ijms-24-10005-s001.zip › Figure S2 Manhattan plot for each chromosome in Shilin..png]

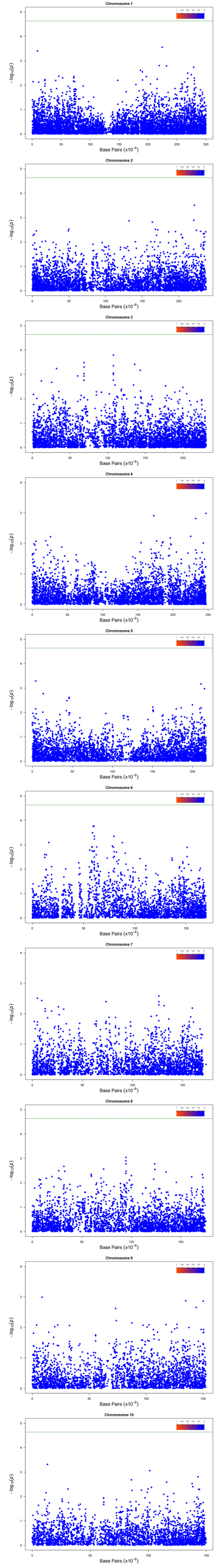

Supplement: Supplementary file 1 [file ijms-24-10005-s001.zip › Figure S3 Manhattan plot for each chromosome in Mojiang..png]

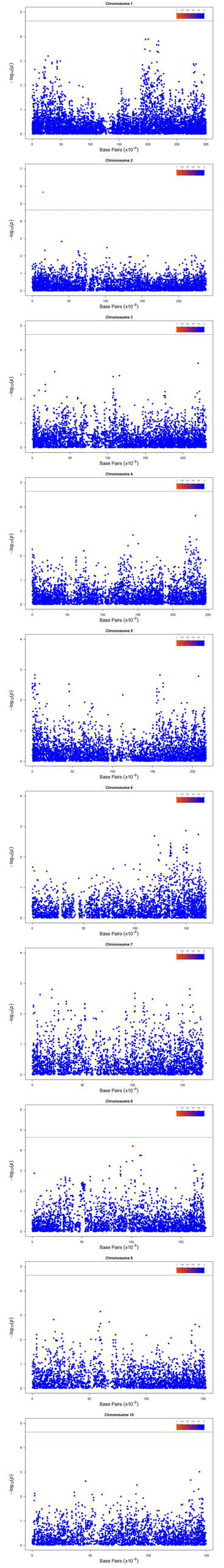

Supplement: Supplementary file 1 [file ijms-24-10005-s001.zip › Figure S4 Manhattan plot for each chromosome in Wenshan..png]

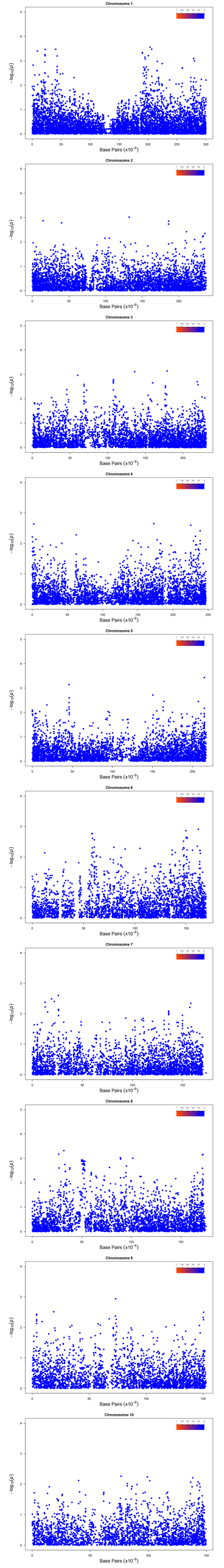

Supplement: Supplementary file 1 [file ijms-24-10005-s001.zip › Figure S5 Manhattan plot for each chromosome in joint environments.png]
